# Childhood contact with social services, self-harm and suicidal or self-harm ideation in young adulthood: a population-wide record-linkage study

**DOI:** 10.1017/S204579602400088X

**Published:** 2025-01-13

**Authors:** S. McKenna, D. O’Reilly, E. Ross, A. Maguire

**Affiliations:** Centre for Public Health, Administrative Data Research Centre Northern Ireland (ADRC NI), Queen’s University Belfast, Belfast, Northern Ireland

**Keywords:** adult outcomes, children’s social care, record-linkage, self-harm, suicidal ideation

## Abstract

**Background:**

Childhood contact with social services is associated with a range of adverse mental health outcomes across the life course, yet there is limited evidence in relation to self-harm and suicidal or self-harm ideation.

**Aims:**

Determine the association between all tiers of childhood contact with social services and presentation to an emergency department (ED) with self-harm or thoughts of suicide or self-harm (ideation) in young adulthood.

**Methods:**

This retrospective cohort study linked population-wide administrative data on self-harm and ideation presentations recorded in the Northern Ireland Registry of Self-Harm (NIRSH) between 2012 and 2015 to primary care registrations and children’s social care data. Multilevel logistic regression models estimated the association between level of contact with social services in childhood (no contact; referred but assessed as not in need; child in need and child in care) and ED-presenting self-harm or ideation in young adulthood.

**Results:**

There were 253,495 individuals born 1985–1993 with full data, alive and resident in Northern Ireland during 2012–2015 (ages 18–30 years). Of all young adults that presented to EDs with self-harm or ideation, 40.9% had contact with social services in childhood. Young adults with a history of care had 10-fold increased odds of self-harm or ideation (OR = 10.49 [95% CI, 9.45–11.66]) relative to those with no contact. Even those assessed as not in need of any help or support in childhood were three times more likely to present with self-harm or ideation (OR = 3.45 [95% CI, 3.07–3.88]).

**Conclusions:**

Understanding the magnitude of childhood adversity amongst adults that present to EDs with self-harm or ideation may inform clinicians’ understanding and therapeutic decision-making. Whilst EDs provide an important setting in which to administer brief interventions, a multi-agency approach is required to reduce self-harm/ideation in young adults that had contact with social services in childhood.

## Introduction

### Background

Self-harm and suicide pose serious public health concerns, with suicide accounting for at least 700,000 deaths per year globally and previous self-harm (intentional self-injury or poisoning regardless of suicidal intent) or ideation (thoughts of suicide-related behaviour) both being strong predictors of death by suicide (Knipe *et al.*, 2022; Ross *et al.*, 2023a, 2023b). The targeted reduction of self-harm as a key precursor to death by suicide is central to national suicide prevention strategies (Department of Health, 2019). Self-harm and ideation represent a substantial number of all hospital emergency department (ED) visits (Clements *et al.*, 2016), therefore EDs present a key entry point for intervention and prevention (Bird *et al.*, 2021). Rates of hospital presenting self-harm and ideation are increasing, especially in adolescents and young adults (Bommersbach *et al.*, 2023; Griffin *et al.*, 2018; Stapelberg *et al.*, 2020). However, little is known on how risk is patterned by childhood contact with social services.

### Existing evidence

Children in contact with social services (i.e. children subject to a referral, in-home child protection/family support measures or out-of-home care) are at increased risk of a range of adverse outcomes given their exposure to adverse childhood experiences (ACEs). Exposure to maltreatment, poverty, family dysfunction and parental mental ill-health or substance misuse are well-established risk factors for self-harm and suicidal ideation (hereafter ideation) (Hawton *et al.*, 2012; Nelson *et al.*, 2020). Research suggests that the prevalence of self-harm and ideation is disproportionately high in children known to social services (Leckning *et al.*, 2021; O’Hare *et al.*, 2023), particularly those placed in out-of-home care (also described as public or state care, or being looked after, hereafter ‘care’) (Allik *et al.*, 2022; Evans *et al.*, 2017; Fleming *et al.*, 2021; McKenna *et al.*, 2023a). Childhood disadvantage can persist into adulthood and contact with social services is associated with a range of adverse health and social outcomes in adulthood (McKenna *et al.*, 2023b; Sariaslan *et al.*, 2022; Xie *et al.*, 2021). However, the association between childhood contact with social services and self-harm and ideation in adulthood has yet to be robustly explored (McKenna *et al.*, 2021).

The majority of prior research is limited by small sample sizes (Courtney *et al.*, 2011; Dixon *et al.*, 2006; Hamilton *et al.*, 2015; Vinnerljung *et al.*, 2006b) and cross-sectional studies that utilise retrospective recall of childhood contact with social services (Afifi *et al.*, 2018; Gentil *et al.*, 2021; Patterson *et al.*, 2015; Roos *et al.*, 2014). To date, the evidence base from large-scale prospective studies is restricted to hospitalised suicide attempt, with studies in Sweden showing elevated risk in adults with a childhood history of care or receipt of child welfare services at home (Almquist *et al.*, 2020; Berlin *et al.*, 2011; Vinnerljung *et al.*, 2015, 2006a). There is a need for population-wide longitudinal cohort studies that can quantify the association between receipt of children’s social care services and ED-presenting self-harm and ideation in young adulthood. Furthermore, high thresholds for intervention from statutory social services mean a large proportion of referrals do not result in any service provision (Bunting *et al.*, 2018; UK Government, 2024). Existing research shows individuals referred to social services but assessed as not in need of any help or protection are at increased risk of mental ill-health and death by suicide (McKenna *et al.*, 2023a, 2023b). Consequently, the aim of this study is to quantify the association between all tiers of contact with children’s social services, including those assessed as not in need, and ED-presenting self-harm and ideation in young adulthood using longitudinal, population-wide linked administrative and health data sources.

## Methods

### Patient and public involvement statement

This study is part of a programme of research being produced in partnership with the charity Voice of Young People in Care (VOYPIC) and the Data Research Advisory Group comprised of lived experience experts (care experienced young people) who meet quarterly with the research team to assist with the development of research questions, interpretation of results and dissemination of findings. The results reported in this paper were the focus of two participatory workshops which directly influenced reporting and dissemination in the following ways: (1) incorporation of the positive message that the majority of care experienced young adults do not present to ED with self-harm or ideation; (2) incorporation of the recommendation that care experienced individuals often require additional support; (3) the development of an easy-read summary of the study and infographics which will be promoted on social media post-publication of the peer-reviewed paper.

### Study design

This retrospective cohort study linked several Northern Ireland (NI) population-wide databases: the National Health Applications and Infrastructure Services (NHAIS) database; the Social Services Client Administration and Retrieval Environment (SOSCARE) system; General Register Office (GRO) death records and the Northern Ireland Registry of Self-Harm (NIRSH). Publicly available area-level measures of rurality and deprivation were also included. Linkage was completed within the NI Trusted Research Environment’s Honest Broker Service (HBS) in collaboration with the Administrative Data Research Centre NI (ADRC NI) by matching of anonymised Health and Care Number (HCN), a unique identifier recorded in any interactions with health and social care services in NI. Extracted data maintained patient anonymity by HBS staff removing all identifiable data from the dataset prior to researcher access. All statistical outputs were subject to additional disclosure control measures, including restrictions on cell numbers to protect confidentiality. Reporting follows the Strengthening of the Reporting of Observational Studies in Epidemiology (STROBE) guidelines (von Elm *et al.*, 2008).

### The datasets

NHAIS contains demographic information on all individuals registered with a general practitioner (GP). NI offers free ‘at the point of service’ health and social care, ensuring almost universal registration within the population (Northern Ireland Statistics & Research Agency, 2016). The NHAIS cohort was all individuals alive and resident in NI during follow-up who were born between 1 January 1985 and 31 December 1993 (*N* = 328,694) and aged at least age 18 years in 2012, allowing for their entire childhood social care history to be captured from SOSCARE before outcome measurement from NIRSH began in 2012. Data on age, sex, area-level income deprivation and area of residence were derived from NHAIS. Area of residence and area-level income deprivation were assigned by the data custodians using patient address in 2010 from NHAIS. Area of residence was based on a classification of settlements in NI and grouped into urban (comprising the two largest cities), intermediate and rural (Northern Ireland Statistics & Research Agency, 2015). A measure of disadvantage was extracted from the income deprivation domain of the Northern Ireland Multiple Deprivation Measure (NIMDM), which provides information on the proportion of the population in each area living in households in receipt of income-related benefits and tax credits in 2010 (Northern Ireland Statistics & Research Agency, 2010), and coded into two groups (less deprived and more deprived). In the creation of this large, linked dataset, the data providers allowed for allocation of area-level measures at one time point only. Mortality data from the GRO were used to delineate the cohort denominator.

SOSCARE data from 1985 to 2015 were linked containing the case records of all individual interactions with children’s social services, which in NI are the responsibility of five regional Health and Social Care Trusts (HSCT). Childhood interaction with social services was classified into four mutually exclusive groups, based on highest level: (i) no contact, (ii) referred but assessed as not in need of help or protection, in every interaction with social services (NIN), (iii) child in need (CIN) (i.e. subject to a CIN plan or child protection measures in their own home) and (iv) child in care (CIC) (i.e. foster, kinship, residential care or placed with parent). Although the legal definition of a child in care in the UK is a ‘looked after child’, lived experience experts identified a strong preference for the term child in care. A child in care is by definition also a child in need but they are examined here as a separate group.

The NIRSH is a national surveillance system which records information on all presentations to EDs in NI for acts of self-harm or thoughts of suicide or self-harm, where no physical act has occurred (hereafter ‘ideation’) (Public Health Agency, 2018). Outcome measures comprised individual-level yes/no indicators for presentation to EDs at any point 2012–2015 for each presentation type: (1) self-harm; (2) ideation and (3) a composite indicator for any self-harm or ideation (i.e. individuals in this group may have presented with self-harm, or with ideation, or with both during the follow-up period). Self-harm and ideation outcomes were non-exclusive, therefore individuals with more than one type of presentation are counted in both standalone outcome groups. The composite indicator for any self-harm or ideation counted individuals only once.

### Study cohort

**Figure 1.**
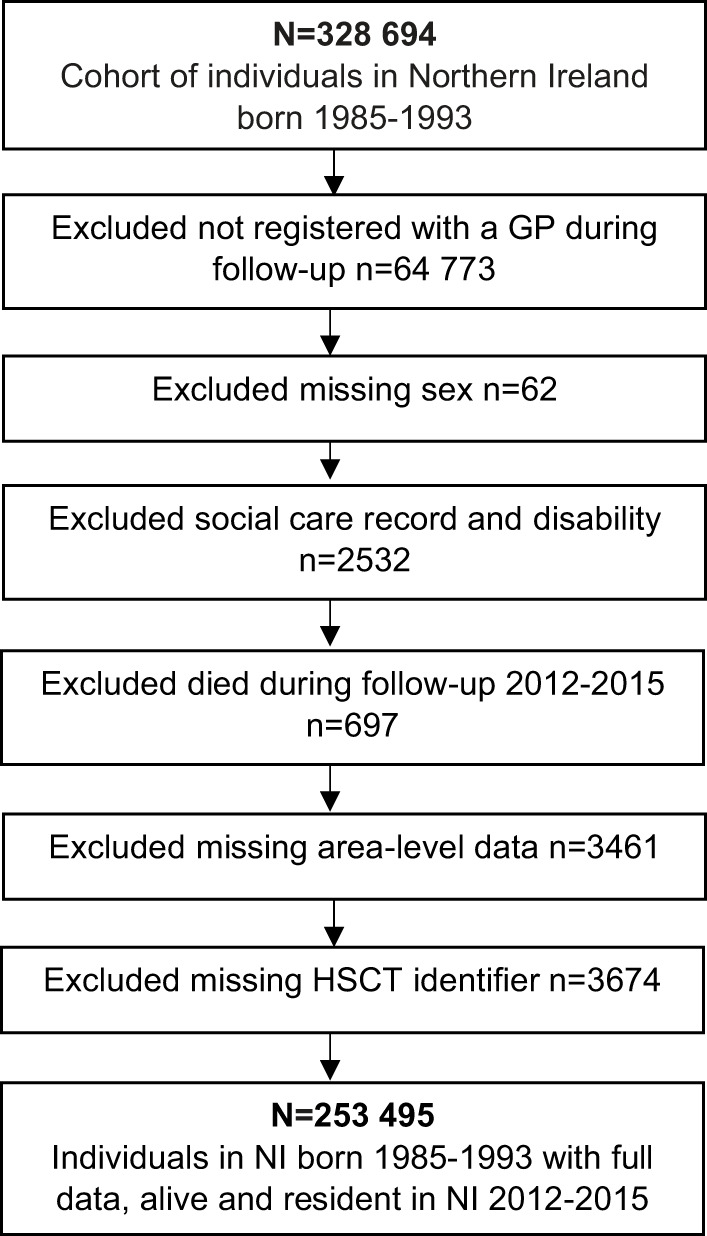
Flowchart of the cohort evolution for the longitudinal analysis of self-harm and ideation in young adulthood based on level of childhood contact with social services.

The final cohort was *N* = 253,495 individuals in NI born 1985–1993 with full data (Fig. 1). The final cohort was aged 18–26 years in 2012 when the NIRSH data (outcome data) began and aged 22–30 years in 2015 when the NIRSH data ended. Individuals not registered with a GP (primarily due to emigration or death) and those who died during follow-up were excluded. Also excluded were individuals missing observations for sex, area-level factors and HSCT identifier to allow complete-case analysis, adjusting for the natural clustering of individuals by HSCT. Individuals recorded within social services data as having a disability were excluded to limit the possible confounding of social care contact and self-harm or ideation risk caused by existing disabilities.

### Statistical analyses

Descriptive statistics were used to delineate cohort characteristics and the prevalence of self-harm and ideation. Outcomes and covariates were compared by social care subgroup using the chi-square statistic for categorical variables. Separate multilevel logistic regression models estimated the association between childhood contact with social services and self-harm and ideation in young adulthood accounting for age, sex, area of residence and deprivation, and the amount of variation attributable to clustering by HSCT. The data are inherently hierarchical, as individuals are naturally clustered within HSCT. Outcomes for two observations in the same cluster are often more alike than are outcomes for two observations from different clusters, even after accounting for individual characteristics. Multilevel modelling (MLM) is an advanced statistical technique for analysing clustered data which reduces the risk of bias resulting from within-cluster homogeneity compared to traditional regression methods.

## Results

Of the cohort of *N* = 253,495 individuals born 1985–1993 and aged 18–26 years at the start of follow-up (1 January 2012), 27,304 (10.8%) had a childhood history of contact with social services (2.9% NIN, 6.5% CIN and 1.4% CIC (Table 1)). There was no substantial difference in sex distribution across categories except for those deemed NIN, which had a male majority (58.8%, *p* < 0.001). The age profile of the cohort varied according to level of social care contact. Among adults with no contact, 30.3% were aged 18–20 years, while over half (50.6%) of adults with a CIN history were in this youngest age category. Level of income deprivation increased with level of social services contact, with a higher proportion of adults known as a CIN and CIC during childhood living in areas with the greatest income deprivation (59.6% and 62.4% respectively). Young adults with a history of placement in care had a more deprived and urban profile than other subgroups.

**Table 1.** Socio-demographic characteristics of the cohort by level of childhood contact with social services (N = 253,495)

	Total cohort	No contact	Not in Need	Child in Need	Child in Care	*p*
	253,495 (100%)	226,191 (89.2%)	7,373 (2.9%)	16,406 (6.5%)	3,525 (1.4%)	
**Sex**						<0.001
*Female*	125,808 (49.6%)	112,502 (49.7%)	3,036 (41.2%)	8,532 (52.0%)	1,738 (49.3%)	
*Male*	127,689 (50.4%)	113,689 (50.3%)	4,337 (58.8%)	7,874 (48.0%)	1,787 (50.7%)	
**Age at 1^st^ Jan 2012**						<0.001
*18−20 years*	80,855 (31.9%)	68,585 (30.3%)	2,706 (36.7%)	8,299 (50.6%)	1,265 (35.9%)	
*21−23 years*	81,557 (32.2%)	72,386 (32.0%)	2,890 (39.2%)	5,060 (30.8%)	1,221 (34.6%)	
*24−26 years*	91,083 (35.9%)	85,220 (37.7%)	1,777 (24.1%)	3,047 (18.6%)	1,039 (29.5%)	
**Deprivation**						<0.001
*More deprived*	111,320 (43.9%)	95,596 (42.3%)	3,740 (50.7%)	9,784 (59.6%)	2,200 (62.4%)	
*Less deprived*	142,175 (56.09%)	130,595 (57.7%)	3,633 (49.3%)	6,622 (40.4%)	1,325 (37.6%)	
**Area of residence**						<0.001
*Rural*	65,295 (25.8%)	60,250 (26.6%)	1,514 (20.5%)	3,050 (18.6%)	481 (13.7%)	
*Intermediate*	85,311 (33.7%)	73,900 (32.7%)	3,267 (44.3%)	6,796 (41.4%)	1,348 (38.2%)	
*Urban*	102,889 (40.6%)	92,041 (40.7%)	2,592 (35.2%)	6,560 (40.0%)	1,696 (48.1%)	

Data are *n* (%).

*p* value from *χ*^2^ tests for categorical variables.

Overall, 4,888 (1.9%) young adult cohort members presented to EDs with self-harm and/or ideation during follow-up (Table 2). While individuals with childhood social care contact comprised 10.8% of the cohort, they accounted for 40.9%* of young adults that presented with self-harm and/or ideation (Table 2, footnotes). Self-harm and ideation are not mutually exclusive: 4,026 (1.6%) young adult cohort members presented to EDs with self-harm whilst 1,669 (0.7%) presented with ideation (Table 2). The prevalence of self-harm and ideation increased stepwise with level of childhood social care contact. Proportionately, the prevalence of self-harm in care experienced young adults (CIC) was approximately 11 times that observed in those with no social care contact (11.2% vs 1.0%) and approximately 14 times for ideation (5.9% vs 0.4%) (Table 2).

**Table 2.** Likelihood of self-harm or ideation among young adults in Northern Ireland born 1985–1993 by level of childhood contact with social services (*N* = 253,495)

Social care history	Number presented to ED (%)		Unadjusted OR	Adjusted OR^a^
**Self-harm (*n* = 4,026)**				
No contact (*n* = 226,191)	2,343	(1.0%)	1.00	1.00
Not in need (*n* = 7,373)	288	(3.9%)	3.94 (3.47–4.46)	3.50 (3.09–3.98)
Child in need (*n* = 16,406)	1,002	(6.1%)	6.33 (5.87–6.83)	5.49 (5.08–5.94)
Child in care (*n* = 3,525)	393	(11.2%)	11.99 (10.71–13.42)	10.33 (9.22–11.58)
**Ideation (*n* = 1,669)**				
No contact (*n* = 226,191)	934	(0.4%)	1.00	1.00
Not in need (*n* = 7,373)	119	(1.6%)	4.21 (3.47–5.11)	3.65 (3.00–4.44)
Child in need (*n* = 16,406)	407	(2.5%)	6.21 (5.52–6.99)	5.58 (4.94–6.31)
Child in care (*n* = 3,525)	209	(5.9%)	15.29 (13.11–17.85)	13.17 (11.26–15.41)
**Any self-harm or ideation (*n* = 4,888)**				
No contact (*n* = 226,191)	2,889	(1.3%)	1.00	1.00
Not in need (*n* = 7,373)	346	(4.7%)	3.90 (3.47–4.37)	3.45 (3.07–3.88)
Child in need (*n* = 16,406)	1,178	(7.2%)	6.09 (5.68–6.53)	5.33 (4.97–5.74)
Child in care (*n* = 3,525)	475	(13.5%)	12.07 (10.88–13.39)	10.49 (9.45–11.66)

Data are number (%) of individuals presenting to ED within each social care category and odds ratios (ORs) with 95% confidence intervals from multilevel logistic regression models adjusted for clustering by Health and Social Care Trust.

a Adjusted for sex, age (years), area-level income deprivation, area of residence and clustering by HSCT.*Of *n* = 4888 individuals that presented with any self-harm or ideation *n* = 1,999 had contact with social services in childhood ((346 + 1,178 + 475)/4888*100 = 40.9%).

The graded relationship between the level of social services contact and self-harm or ideation is also evident in the multilevel regression analyses (Table 2). Likelihood increased stepwise with level of childhood social care contact with modest attenuation by adding covariates. Care experienced young adults were over 10 times more likely to present with self-harm (OR = 10.33 [95% CI, 9.22–11.58]) and over 13 times more likely to present with ideation (OR = 13.17 [95% CI, 11.26–15.41]) compared to young adults with no contact with social services in childhood. Young adults with a CIN history were over five times more likely to present with self-harm or with ideation (OR = 5.49 [95% CI, 5.08–5.94] and OR = 5.58 [95% CI, 4.94–6.31] respectively) compared to those with no contact with social services in childhood. Even young adults assessed as NIN in childhood were over three times more likely to present with self-harm (OR = 3.50 [95% CI, 3.09–3.98]) or ideation (OR = 3.65 [95% CI, 3.00–4.44]) than those with no contact with social services.

The significant Chi-squared test (MLM vs logistic) in models for likelihood of self-harm (*χ*^2^ = 12.01, *p* < 0.001), ideation (*χ*^2^ = 12.58, *p* < 0.001) and any self-harm or ideation (*χ*^2^ = 12.39, *p* < 0.001) shows that the multilevel models are a better fit to the data than logistic models which do not account for HSCT variation. In fully adjusted models, the intraclass correlation estimated that Trust variation comprised just 0.2% of the total residual variance for self-harm presentations, 0.4% for ideation and 0.2% for any self-harm or ideation [full results available on request].

## Discussion

To our knowledge, this is the first study to capture population-wide, national surveillance data on presentations by young adults to EDs for self-harm or ideation, stratified by all tiers of childhood social care contact. It is the first to quantify that 40.9% of young adults (ranging from 18 to 30 years old) who present to EDs due to self-harm or ideation have at least some contact with social services in childhood. This study extends previous population-wide evidence of ED-presenting suicide attempt among adults with a history of in-home or out-of-home care (Almquist *et al.*, 2020; Berlin *et al.*, 2011; Vinnerljung *et al.*, 2015, 2006a) by including data on all self-harm acts, all ideation and all tiers of childhood contact with social services, including referrals that did not result in service provision. The highest risk is observed in care experienced young adults, who are 10 times more likely to present to an ED with self-harm and 13 times more likely to present with ideation compared to peers with no childhood contact. However, even those deemed not in need were over three times more likely to present with self-harm or ideation. The graded relationship may be indicative of confounding by indication, as level of social care intervention increases with the degree of potential harm to the child. There is a dose–response relationship between number and severity of ACEs and the incidence and persistence of mental health problems in adulthood (Bürgin *et al.*, 2023; Daníelsdóttir *et al.*, 2024).

The large and disproportionate burden of self-harm and ideation in these young adults may be linked to influences before, during and after contact with social services. Children come to the attention of social services for a reason. Exposure to domestic violence and concerns about parental mental health are the most common factors recorded in social care assessments in the UK (UK Government, 2024), which tend to occur in clusters with other types of adversity such as poverty and maltreatment (Sahle *et al.*, 2022; Steptoe *et al.*, 2019). Children in care often experience barriers to accessing appropriate mental health support both during placement and after aging-out (Fargas-Malet and McSherry, 2018; Phillips *et al.*, 2023, 2024). Disadvantage can be reinforced or exacerbated across the life course, resulting in health, social and economic inequalities which are risk factors for self-harm and suicidal behaviour (Brännström *et al.*, 2017).

While likelihood of self-harm or ideation is highest in care experienced young adults, these represent a comparatively small exposure group and three times as many adults with lower tiers of social care contact presented to EDs with self-harm or ideation. The significant overlap between childhood contact with social services and need for acute care for self-harm or suicidal crisis requires population-level strategies to reduce risk factors and evidence-based practices targeted at individuals. The National Institute for Health and Care Excellence (NICE) guidance for self-harm emphasises the need for a psychosocial assessment following any self-harm episode (National Institute for Health and Care Excellence, 2022) and liaison psychiatry teams have a crucial role delivering psychosocial assessment (Mughal *et al.*, 2023). However, there is considerable variation in the management of self-harm in the ED setting (Arensman *et al.*, 2018) and there are currently no NICE guidelines covering the management of ideation. New models of care including a national clinical programme for the management of self-harm and suicidal ideation (NCPSHI) in hospital in Ireland (Cully *et al.*, 2023; Health Service Executive Ireland, 2022) and the Emergency Department Safety Assessment and Follow-up Evaluation 2 (ED-SAFE 2) cluster RCT (Boudreaux *et al.*, 2023) demonstrate improved rates of psychosocial assessment and mental health referrals following discharge and reduced presentations for suicidal behaviour. While interventions should not be restricted to high-risk groups, clinical training and guidelines could assist clinicians to identify individuals at increased risk due to their social care history.

### Strengths and limitations

A major strength of this study was the use of longitudinal, population-wide administrative data that captured all tiers of childhood contact with social services, including those assessed as not in need. Outcome data were accessed via the only national surveillance system for hospital presenting self-harm and ideation that exists worldwide. However, there are also several limitations of note. As with all observational studies causation cannot be inferred and future studies with designs suited to establish causal inferences are needed. As the study was restricted to ED-presenting self-harm and ideation, results may not be generalisable to individuals in the community who self-harm or have suicidal thoughts. We were limited with the data available and did not have access to child or adult mental health services data which may explain a significant proportion of the likelihood of the outcome. In addition, children’s social care systems and populations vary internationally, and NI has the highest rate of ED-presenting self-harm in the United Kingdom, warranting the replication of these findings in other jurisdictions. The exclusion of individuals that died before the study end date, particularly deaths by suicide, may introduce survival bias and the potential underestimation of self-harm or ideation risk associated with childhood social care contact.

## Conclusion

Mental ill-health is a growing public health concern and whilst self-harm and ideation themselves are behaviours that require appropriate support, they are also the largest known predictors of death by suicide. Understanding who is most at risk and who may benefit from targeted interventions is vital to improving mental health outcomes, reducing rates of death by suicide and understanding prevention pathways. Most children in contact with social services do not present to an ED with self-harm or ideation in young adulthood. However, the large and disproportionate burden of self-harm and ideation within this group underscores the need for a policy response and is a prime opportunity for interventions targeted at young adults with a childhood history of contact with social services or with children still in contact with social services or approaching leaving care.

This study highlights two things. The first is the complex background of over 40% of young adults who present to EDs with self-harm or ideation. Understanding the magnitude of childhood adversity amongst this population may help inform health professionals’ and clinicians’ understanding and therapeutic decision-making. The findings warrant consideration during the development or review of clinical guidelines which identify groups and communities that have increased risk of self-harm or ideation and specify models of care to respond to their specific needs following presentation to ED (Health Service Executive Ireland, 2022). Engagement with social services and individuals with lived experience of social care and self-harm or ideation is also recommended as part of this process. The second is the need for multi-agency care. Whilst EDs provide an important setting in which to provide psychosocial assessment and administer brief interventions to individuals who present with self-harm or ideation, early intervention and increased mental health support services for adults that had contact with social services in childhood requires a multi-agency approach with health and social services.

The need to improve young people’s mental health has been a key element of recent independent reviews of children’s social care in England (MacAlister, 2022), Scotland (Duncan, 2020) and NI (Jones, 2023). The NI Review recommended the need for the development of emotional health and well-being services within Social Services separate from clinical Child and Adolescent Mental Health Services (CAMHS) (Jones, 2023). This study provides evidence to support this recommendation as early intervention may be key to preventing these individuals presenting to EDs with self-harm or ideation in young adulthood. The authors would suggest extending the provision of these services to all levels of contact with social services and not just limit them to children in care as the largest absolute number of young adults presenting to ED with self-harm or ideation come from lower tiers of social services intervention. The Review also recommended the expansion of post-18 years support (Jones, 2023). This study illustrates the continued increase in likelihood of self-harm and/or ideation amongst young adults known to social services in childhood, suggesting the need for ongoing support and in particular mental health support after ageing out of the children’s social care system.

## Data Availability

Data collected by the Northern Ireland Registry of Self-Harm are confidential and cannot be made available publicly. Access to data is available via the Northern Ireland Trusted Research Environment’s Honest Broker Service for Health and Social Care (HSC) Northern Ireland, once approval from all Health and Social Care Trusts in Northern Ireland has been received. To access data from the Northern Ireland Registry of Self-harm, please contact the Public Health Agency (reception.pha@hscni.net). NHAIS and SOSCARE data are available for research projects in the public interest that relate to Health and Social Care, subject to application and approval by the Honest Broker Service Governance Board (for information contact honestbrokerservice@hscni.net).
